# Bone Mineral Density in Cystic Fibrosis Patients with the CFTR I1234V Mutation in a Large Kindred Family Is Associated with Pancreatic Sufficiency

**DOI:** 10.1155/2014/465395

**Published:** 2014-06-30

**Authors:** Atqah Abdul Wahab, M. Hammoudeh, Mona Allangawi, Fawziya Al-Khalaf, Prem Chandra

**Affiliations:** ^1^Department of Pediatrics, Hamad Medical Corporation, Doha, Qatar; ^2^Weill Cornell Medical College, P.O. Box 3050, Doha, Qatar; ^3^Department of Internal Medicine, Hamad Medical Corporation, Doha, Qatar; ^4^Medical Research Center, Hamad Medical Corporation, Doha, Qatar

## Abstract

*Objectives*. To study bone mineral density (BMD) in cystic fibrosis (CF) children and adults with the CFTR I1234V mutation associated with pancreatic sufficiency. *Methods*. Lumbar spine, total hip, and whole-body mineral density were measured by dual-energy radiographic absorptiometry (DEXA) scan. *Z* score was used for those less than 21 years and *T* score was used for those 21 years or older. *Results*. Twenty-one CF patients were younger than 21 years and 5 CF patients were 21 years or older. Mean age was 17.29 ± 4.95 years, ranging from 10 to 33 years. The mean BMD *Z* scores for patients younger than 21 years were −0.69 ± 0.96 (lumbar spine = L1–L4), −0.48 ± 0.92 (total hip), and −0.38 ± 0.86 (total body). The mean *T* scores for patients 21 years or older were 0.14 ± 0.7 (L1–L4), 0.38 ± 1 (total hip), and 0.52 ± 1.03 (total body). BMD reduction less than −1 was found in 7 (26.9%) CF patients. Vitamin D deficiency in 20 CF patients (76.9%) tended to be lower in CF patients with low BMD. BMD was significantly correlated with FEV1; however, no significant association was observed with *P. aeruginosa* colonization. *Conclusion*. BMD reduction does occur in patients with mild CFTR mutation associated with pancreatic sufficiency.

## 1. Introduction

CF is one of the most common inherited diseases among Caucasians [1]. It is caused by mutation in the cystic fibrosis transmembrane conductance regulator (*CFTR*) gene, which encodes a transmembrane glycoprotein [2, 3]. The CF transmembrane conductance regulator has been shown to function as a cyclic adenosine monophosphate- (cAMP-) regulated chloride channel at the apical membrane of epithelial cells [4]. One of the main consequences of mutations in the* CFTR* gene is a dysfunction of ion channels resulting in elevated sweat chloride concentrations, pancreatic insufficiency, and progressive lung disease [5].

Newly introduced therapies and aggressive management have led to a median expected survival age of 36 years [6]. However, new clinical problems that need to be identified and therapeutically addressed may become evident as the population ages. A number of reports have documented CF-related low BMD in both adults and children with CF [7–9]. CF-related bone disease (CFRBD) is multifactorial in etiology, primarily related to imbalanced bone deposition and resorption [10]. Other factors influencing CFRBD include low body mass index, vitamins D and K insufficiency, poor Ca^2+^ absorption and excessive Ca^2+^ secretion in the gastrointestinal tract, low levels of insulin-like growth factor 1, chronic bacterial infection with associated chronic inflammation and heightened cytokine activity, and treatment with antibiotics and glucocorticoids [7, 11]. The prevention, early diagnosis, and treatment of CFRBD are critical because pain, deformity, immobility related to fragility fractures, and kyphosis may contribute to reduction in lung function and effectiveness of cough [12].

BMD is commonly assessed using DEXA and is reported as both a* T* score and a* Z* score. Low BMD is defined using WHO criteria which state that a* T* score between −1.0 and −2.5 is osteopenia and <−2.5 is considered osteoporosis. The* T* score refers to the individuals' BMD compared to individuals of the same gender between the ages of 20–40 years. The* Z* scores refer to the number of standard deviations between a patient BMD value and the average value of an age and gender matched healthy control population. A patient with a BMD* Z* score below −2 is considered to have CF-related low BMD (Cystic Fibrosis Trust, 2007) [13].

The CFTR I1234V mutation is one of the common CF mutations among Arabs in the Gulf region belonging to a large kindred Arab tribe [14, 15]. The BMD in these CF patients with CFTR I1234V mutation has not been studied. The aim of this study was to study the spectrum of BMD in a cohort of CF patients greater than 10 years of age. We evaluate the relationship between BMD lumbar spine, total hips, and whole-body mineral content and age, sex, BMI, serum 25[OH]D, and severity of illness by chronic* Pseudomonas aeruginosa* (*P. aeruginosa*) colonization and lung function.

## 2. Methods

This study recruited thirty-three CF patients having the CFTR I1234V mutation that were more than 10 years old who attended the CF Clinic at Hamad Medical Corporation, Doha, Qatar, between November 2009 and April 2010. Seven patients did not show up in follow-up CF Clinic and their BMD values could not be recorded and hence were excluded from the statistical analysis. This study was initiated as a pilot study and therefore there was no formal sample size calculation done for this study. CF diagnosis was established by documentation of elevated sweat chloride levels and/or assessment of two CFTR gene mutations. None had been acutely ill for at least four weeks and had taken oral, intravenous, or inhaled steroids. None had taken vitamin K or D supplements in three months prior to the study.

This cross-sectional study was approved by the Research Ethics Committee at Hamad Medical Corporation. Written informed consent was obtained from the parents or legal guardians of subjects less than 18 years old and from subjects aged 18 years or older.

### 2.1. Anthropometric and Clinical Parameters

Heights were measured using a Harpenden stadiometer and height* Z* score was calculated using standard formula for calculating* Z* score. Anthropometric measurements were performed using digital electronic platform scale and standing height measurement without shoes and with the patient being lightly dressed using a stadiometer. Body mass index (BMI) was calculated by dividing weight in kg by height squared in meters {weight (Kg)/(height (m))^2^}. BMI* Z* score was calculated and adjusted for age and gender. Puberty was evaluated with Tanner staging. Subjects performed spirometric tests in the respiratory laboratory unit in accordance with the standards of the American Thoracic Society [16] using reference value for spirometry in children and adolescents given by Knudson et al. [17]. The best recorded forced expiratory volume in 1 second (FEV1) using a flow-sensing spirometer (Sensor Medicus Model V6200, Germany) was recorded.

### 2.2. Laboratory Measurements

A single venous blood sample was obtained from each patient for measurement of serum calcium, phosphorus, alkaline phosphatase, albumin, and liver enzymes using a Roche modulator analyzer. Circulating concentrations of 25[OH]D were measured using DiaSorin 25[OH]D radioimmunoassay kits double antibody assay (DiaSorin, Inc., Stillwater, Minnesota, USA). Measurement of vitamin K levels was performed in France using high-performance liquid chromatography (HPLC). Each measurement was categorized as normal or abnormal on the basis of standard age-appropriate limits as defined by Hamad Medical Corporation laboratory.

### 2.3. Bone Densitometry Measurements

Bone mineral density of the lumbar spine (L1–L4), total hip, and the total body was determined by DEXA by Lunar Prodigy system (Lunar Corporation, Minnesota, NJ, USA). Bone densities were expressed as bone mineral density (BMD, g/cm^2^). Two sites were measured, namely, the lumbar spine (L1–L4) anteroposteriorly and the average of femur. All measurements were performed at the BMD Unit at HMC by a single experienced technologist and reviewed by one of the authors (M. H.).

The normal BMD data base for children was used to derive the* Z* score and the adult data base to derive the* T* score. The BMD machine in our institution is programmed to use the* Z* score for those who are less than 21 years using Lunar Australian Pediatric Norms and* T* score is used for those who are 21 years or older using Lunar Middle East Norms. The quality control of the DEXA scan was monitored daily. The precision of the system was assessed by duplicate measurements of 15 individuals aged 10–26. The precision error was 1.8% for the spine and 1.4% for the total femur.

### 2.4. Statistical Analysis

Categorical and continuous values were expressed as frequency (percentage), mean ± SD, median, and range. Descriptive statistics were used to summarize all demographic and other characteristics of the participants. Quantitative variables means between the two independent groups were analyzed using unpaired *t*-test and Mann-Whitney *U* test. Associations between two or more qualitative or categorical variables were assessed using chi-square test. Chi-square test with continuity correction factor and Fisher's exact test were used in case of small cell frequencies. Associations between specific variables including the age and BMD* Z* and/or* T* scores were examined using Pearson's correlation coefficients. Multiple linear regression analysis was applied to assess and examine the effect of different covariates such as age, gender, height* Z* score, BMI* Z* score, multivitamins, serum 25[OH]D, vitamin K, chronic* P. aeruginosa* colonization, and FEV1 on outcome variable BMD* Z* scores and/or* T* scores. A two-sided *P* value <0.05 was considered to be statistically significant. All statistical analyses were done using the statistical package SPSS 19.0 (SPSS Inc., Chicago, IL).

## 3. Results

Thirty-three CF patients with I1234V CFTR from a single large Arab kindred tribe were approached to participate in this cross-sectional study. Seven patients did not show up in follow-up CF Clinic and their BMD values could not be recorded and hence were excluded from the statistical analysis. Consequently twenty-six CF patients with CFTR I1234V (16 males and 10 females) from 14 families were enrolled in this study. There were three CF sibling pairs, three families with three CF siblings, and one family with four siblings. Twenty-one CF patients were younger than 21 years and 5 CF patients were 21 years of age or older. The mean age (± standard deviation) of the group was 17.29 ± 4.95 years ranging from 10 to 33 years. All the patients were pancreatic sufficient. The majority of patients were not taking nutritional supplements or multivitamins. Seven patients (26.95%) were exposed to sun for more than 30 minutes per day. Sixteen CF patients (61.5%) were colonized with* P. aeruginosa*. None of CF patients had evidence of clinical pubertal delay.  Table 1 summarizes baseline demographic, anthropometric, nutritional, and other clinical characteristics.

Serum calcium, phosphorus, alkaline phosphatase levels, vitamin K, albumin, and liver enzymes were all within the normal range. Twenty CF subjects (76.9%) had levels of 25[OH]D below the normal levels (<30 ng/mL) and 6 CF patients had normal 25[OH]D levels. None of our patients had a history of bone fracture or pubertal delay. Mean BMD* Z* scores in the younger group were −0.69 ± 0.96 (L1–L4), −0.48 ± 0.92 (total hip), and −0.38 ± 0.86 (total body) demonstrating more deficits at the lumbar spine. Mean BMD* T* scores for patients 21 years or older were 0.14 ± 1.13 (L1–L4), 0.38 ± 1 (total hip), and 0.52 ± 1.03 (total body).

BMD reduction less than −1 was found in 7 (26.9%) CF patients. Two of seven had BMD* Z* or* T* scores below −2. Six of the 7 CF patients with low BMD were younger than 21 years at one or more sites, the youngest being 14 years old. BMI was significantly lower among CF patients with low BMD (17.9 ± 1.3 versus 23.7 ± 6.5; *P* = 0.033). Vitamin D deficiency was found in 20 CF patients (76.9%) with a mean of 25[OH]D tending to be lower in CF patients with low BMD.

**Figure 1:**
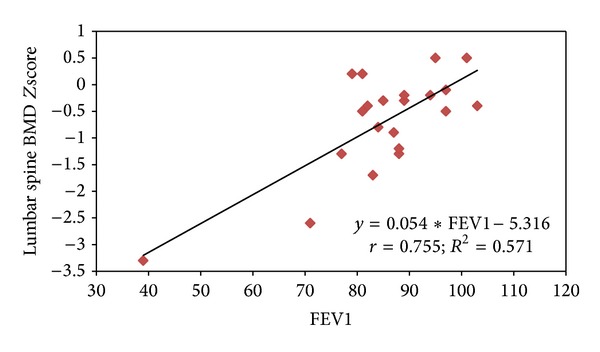
Relationship between lumbar spine BMD* Z* score and FEV1.

**Figure 2:**
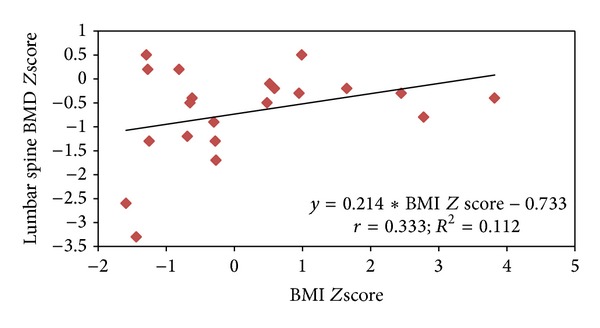
Relationship between lumbar spine BMD* Z* score and BMI* Z* scores.

Table 2 demonstrated the association of different factors with vitamin D deficiency. Mean ALP was found to be significantly higher in the vitamin D deficient group compared to normal vitamin D levels (122.9 ± 53.2 versus 80.7 ± 23.0; *P* = 0.011). Similarly, mean FEV1 and BMI were observed to be higher in the vitamin D deficient group compared to normal vitamin D levels; however, the difference did not achieve statistical significance (*P* > 0.05). Among the patients younger than 21 years, FEV1 was significantly and positively correlated with lumbar spine BMD* Z* scores (*r* = 0.755; *P* < 0.001), total hip (*r* = 0.672; *P* < 0.001), and whole body (*r* = 0.736; *P* < 0.001). Lumbar spine BMD* Z* scores were positively correlated with BMI* Z* scores, despite being not significant statistically (*r* = 0.333; *P* > 0.05) (Figures 1 and 2). Among females mean lumbar spine BMD* Z* scores were found to be higher compared to males (−0.51 ± 0.62 versus −0.86 ± 1.19; *P* = 0.621). There was no significant association observed between BMD* Z* score and age, gender, height* Z* scores,* Pseudomonas aeruginosa* (*P. aeruginosa*) colonization, 25[OH]D levels, multivitamin, phosphorus, and ALP (*P* > 0.05) (data not shown in the table).

Multiple linear regression analysis was used to assess the effect of age, gender, height* Z* score, BMI* Z* score, multivitamins, serum 25[OH]D, chronic* P. aeruginosa* colonization, and FEV1 on outcome variable BMD* Z* scores and showed that FEV1 was significantly and positively associated with lumbar spine BMD* Z* scores (regression coefficient = 0.755; *P* < 0.001), total hip* Z* scores were significantly associated with FEV1 (regression coefficient = 0.522; *P* = 0.004) (regression coefficient = 0.419; *P* = 0.017), and whole-body* Z* scores were significantly associated with FEV1 (regression coefficient = 0.514; *P* = 0.001) and BMI* Z* scores (regression coefficient = 0.382; *P* = 0.007).

## 4. Discussion

In this study we observed low BMD during the first two decades of life in CF cohorts with CFTR I1234V mutation associated with pancreatic sufficiency. BMD* Z* score was significantly and positively associated with FEV1; however, no significant association was observed with BMI in CF patients. In a study by Gronowitz et al. [18] reported BMD* Z* scores were significantly lower in CF patients compared to normal population despite normal anthropometry and the strongest correlation was found with lung function. Donadio et al. [19] reported that most CF patients had BMD within normal limits and presented a positive correlation with pulmonary function and negatively correlated with chronological age and age at diagnosis. However, in our study there was no significant association observed between BMD and chronic* P. aeruginosa* colonization. Data on association of CFRBD and nutritional status have been conflicting. A recent study suggested that the origin of CF bone disease in early childhood may be independent of nutritional status or disease severity [20]. In contrast, another study suggested that BMD may be reduced and related to malnutrition and severity of disease, with males being more vulnerable [21].

Vitamin D plays a critical role in bone health by enhancing intestinal absorption of calcium and regulating bone turnover. The most striking finding in our study was that subjects with CF had significantly low serum 25[OH]D. About 76.9% of our patients with CF had 25[OH]D levels below 30 ng/mL, the suggested cutoff proposed by the Consensus Conference of the Cystic Fibrosis Foundation. Recently, we have reported a high prevalence of vitamin D deficiency despite normal pancreatic exocrine function, which might be related to the hot climate of the Arabian Gulf region and the usual traditional cloth that covers most of the body, and, in addition, they stay mainly indoors and may not have much adequate direct exposure to sun [22]. In agreement with the recent study, it has been reported that inefficient levels of vitamin D are common and contribute significantly to impaired bone health and underline the need for higher supplementation doses in CF patients [23]. In another study the result of an audit of DEXA scan was reported for 108 adults with CF; the most common risk factors for bone loss were vitamin D deficiency (89%), low body mass (39%), and post-lung transplantation (25%) [22]. However, there are conflicting reports regarding the correlation between 25[OH]D levels and decreased BMD [20–22]. Closer monitoring of vitamin D status in CF patients may be warranted because appropriate interventions at an early age may decrease the prevalence and severity of bone disease later in life. Vitamin K is thought to play an important role in bone formation [24]. None of our patients had a vitamin K deficiency. Vitamin K deficiency is seen in 40% of patients with CF who are receiving fat-soluble vitamin supplementation [24].

Some limitation of the study must be stressed. The study was cross-sectional with a small cohort from whom we cannot determine the mechanisms resulting in CFRBD. Seven CF patients with CFTR I1234V mutations did not show up in follow-up CF Clinic and their BMD values could not be obtained and hence were excluded from the analysis. Another limitation is that our BMD machine uses Australian Pediatrics Norms as reference standard while for adults it uses Lunar Middle East Norms which could have contributed to possible bias towards lower values in children younger than 21 years. However, we would like to stress the fact that the main objective of the study was not to compare the two groups together but to compare the BMD with lung function and the severity of illness by chronic* P. aeruginosa* colonization.

## 5. Conclusions

BMD reduction does occur in CF patients with mild CFTR mutation associated with pancreatic sufficiency. The results of our study suggest that pulmonary function (FEV1) and related factors might be the main determinants of BMD in CF. We recommend that all children and adults with CF undergo an assessment of BMD and body composition early in their life to make it possible to target those who need preventive treatment. Follow-up data as a longitudinal study with the larger cohort of the mutation CFTR I1234V in the Gulf region is strongly recommended to develop effective preventive treatment and programs.

## Figures and Tables

**Table 1:** Baseline demographic, anthropometric, nutritional, and other clinical characteristics.

Characteristics	Mean ± SD [median (min–max)] *N* (%)
Age (years)	17.3 ± 4.9 [16.9 (10–33)]
Height (cm)	159.2 ± 11.85 [160 (133–178)]
Weight (kg)	56.8 ± 19.42 [53 (26–95)]
Body mass index (BMI)	22.1 ± 6.2 [19.7 (14.9–36.7)]
Phosphorus (mmol/L)	1.3 ± 0.20 [1.4 (1–1.7)]
Calcium level (mmol/L)	2.3 ± 0.08 [2.3 (2.2–2.5)]
ALP (U/L)	113.2 ± 50.9 [92.5 (58–225)]
25[OH]D levels (ng/mL)	21.6 ± 9.6 [22 (6–42)]
Vitamin K levels (ng/L)	222.5 ± 136.8 [181 (107–585)]
FEV1	82.9 ± 14.7 [86 (39–103)]
L1–L4 BMD *Z* score	−0.69 ± 0.96 [−0.4 (−3.3–0.5)]
Total hip *Z* score	−0.48 ± 0.92 [−0.2 (−2.3–0.9)]
Total body *Z* score	−0.38 ± 0.86 [−0.2 (−2.5–1)]
L1–L4 BMD *T* score	0.14 ± 1.13 [0.7 (−1.8–0.9)]
Total hip *T* score	0.38 ± 1.0 [0.6 (−1.3–1.4)]
Total body *T* score	0.52 ± 1.03 [0.6 (−1.1–1.7)]
Gender	
Male	16 (61.5%)
Female	10 (38.5%)
Chronic *P. aeruginosa *	
Yes	16 (61.5%)
No	10 (38.5%)
Multivitamin	
Yes	6 (23.1%)
No	20 (76.9%)
Time of exposure to the sun	
>30 min per day	7 (26.9%)
≤30 min per day	19 (73.1%)
25[OH]D levels	
≥30 (ng/mL)	6 (23.1%)
<30 (ng/mL)	20 (76.9%)

*T* score was calculated for a patients' age more than 21 years.

**Table 2:** Association of different factors between CF patients having normal and low vitamin D [25(OH)D].

Characteristics	CF patients with [25(OH)D]≥30 (ng/mL) (*n* = 6)	CF patients with [25(OH)D] <30 (ng/mL) (*n* = 20)	*P* value
Age (years)	17.3 ± 2.7	17.3 ± 5.5	0.999
BMI	19.6 ± 2.9	22.9 ± 6.7	0.099
Gender (female)	3 (50%)	13 (65%)	0.644
*Pseudomonas* (+ive)	6 (100%)	10 (50%)	**0.053**
Time of exposure to the sun (<30 min per day)	3 (50%)	16 (80%)	0.293
Multivitamin (no)	2 (33.3%)	18 (90%)	**0.013**
Phosphorus (mmol/L)	1.3 ± 0.17	1.4 ± 0.20	0.504
Calcium level (mmol/L)	2.3 ± 0.09	2.3 ± 0.08	0.730
ALP (U/L)	80.7 ± 23.0	122.9 ± 53.2	**0.011**
Vitamin K levels (ng/L)	306.2 ± 170.6	187.6 ± 110.02	0.105
FEV1	75.3 ± 26.2	85.2 ± 8.9	0.403
L1–L4 BMD *Z* score	−0.58 ± 1.41	−0.52 ± 0.92	0.898
Total hip *Z* score	−0.13 ± 1.21	−0.38 ± 0.93	0.607
Total body *Z* score	−0.18 ± 1.37	−0.22 ± 0.83	0.944

BMI: body mass index.
